# Classification of psychiatric symptoms using deep interaction networks: the CASPIAN-IV study

**DOI:** 10.1038/s41598-021-95208-y

**Published:** 2021-08-03

**Authors:** Hamid Reza Marateb, Zahra Tasdighi, Mohammad Reza Mohebian, Azam Naghavi, Moritz Hess, Mohammad Esmaiel Motlagh, Ramin Heshmat, Marjan Mansourian, Miguel Angel Mañanas, Harald Binder, Roya Kelishadi

**Affiliations:** 1 Biomedical Engineering Department, Engineering Faculty, University of Isfahan, Isfahan, 81746-73441 Iran; 2 Biomedical Engineering Research Centre (CREB), Automatic Control Department (ESAII), Universitat Politècnica de Catalunya-Barcelona Tech (UPC), Building H, Floor 4, Av. Diagonal 647, 08028 Barcelona, Spain; 3 Epidemiology and Biostatistics Department, Health School, Isfahan University of Medical Sciences, Isfahan, 81746-73461 Iran; 4 Department of Electrical and Computer Engineering, University of Saskatchewan, Saskatoon, SK S7N 5A9 Canada; 5 Department of Counseling, Faculty of Education and Psychology, University of Isfahan, Azadi Sq, Isfahan, 8174673441 Iran; 6 Faculty of Medicine and Medical Center, University of Freiburg, 79104 Freiburg, Germany; 7 Department of Pediatrics, Ahvaz Jundishapur University of Medical Sciences, Ahvaz, 61357-15794 Iran; 8 Chronic Diseases Research Center, Endocrinology and Metabolism Population Sciences Institute, Tehran University of Medical Sciences, Tehran, Iran; 9 Pediatric Cardiovascular Research Center, Isfahan Cardiovascular Research Institute, Isfahan University of Medical Sciences, 81746-73461 Isfahan, Iran; 10 Biomedical Research Networking Center in Bioengineering, Biomaterials, and Nanomedicine (CIBER-BBN), 28029 Madrid, Spain; 11 Pediatrics Department, Child Growth and Development Research Center, Research Institute for Primordial Prevention of Non-Communicable Disease, Isfahan University of Medical Sciences, Isfahan, Iran

**Keywords:** Biomedical engineering, Psychiatric disorders, Risk factors, Paediatric research, Psychology

## Abstract

Identifying the possible factors of psychiatric symptoms among children can reduce the risk of adverse psychosocial outcomes in adulthood. We designed a classification tool to examine the association between modifiable risk factors and psychiatric symptoms, defined based on the Persian version of the WHO-GSHS questionnaire in a developing country. Ten thousand three hundred fifty students, aged 6–18 years from all Iran provinces, participated in this study. We used feature discretization and encoding, stability selection, and regularized group method of data handling (GMDH) to classify the a priori specific factors (e.g., demographic, sleeping-time, life satisfaction, and birth-weight) to psychiatric symptoms. Self-rated health was the most critical feature. The selected modifiable factors were eating breakfast, screentime, salty snack for depression symptom, physical activity, salty snack for worriedness symptom, (abdominal) obesity, sweetened beverage, and sleep-hour for mild-to-moderate emotional symptoms. The area under the ROC curve of the GMDH was 0.75 (CI 95% 0.73–0.76) for the analyzed psychiatric symptoms using threefold cross-validation. It significantly outperformed the state-of-the-art (adjusted *p* < 0.05; McNemar's test). In this study, the association of psychiatric risk factors and the importance of modifiable nutrition and lifestyle factors were emphasized. However, as a cross-sectional study, no causality can be inferred.

## Introduction

Mental health and illness are a public health concern^1^. Nowadays, the prevalence of non-communicable diseases (NCDs), such as mental disorders, is rapidly increasing, and the prevention of their associated risk factors has been one of the world's health priorities^2^. The World Health Organization (WHO) demonstrated that in different age groups, about 450 million people are suffering from severe and mild mental disorders worldwide^3^. Moreover, at least 52 million people with severe mental disorders, such as schizophrenia, and nearly 150 million people tolerate unspecified mental diseases, including psychological distress^4^. The literature also discussed whether such distress is a symptom of a mental disorder or a marker of functional impairment^5^.

Psychological distress is the most common mental health issue affecting many children and is considered one of the leading causes of the global burden of disease^6^. The National Comprehensive Cancer Centre (NCCN) defines distress as an unpleasant emotional experience of a psychological problem, such as depression, worriedness, and panic^7^. If children's psychological distress remains untreated, their development is significantly influenced.

Children worldwide are affected by similar psychological distress as adults^8^. It is related to an increased risk of harmful events, including drug addiction and poor educational performance^9^. They are common in the Eastern Mediterranean Region (EMR), including Iran and neighboring countries, and are the leading cause of years of life lived with disability (YLDs). In EMR, depression was accounted for the most Disability-Adjusted Life Years (DALYs), and worriedness ranked second in 2013^10^. In summary, depression, and worriedness, two essential components of psychological distress, are among the illness and disability leading causes in adolescents^11^.

Many studies have attempted to investigate the association between several factors and psychological distress among children. Risk factors associated with such psychological distress appear to be modifiable, partly through the link between these characteristics and lifestyle factors. In general, the literature review shows that the spread of psychological distress changes depending on various factors such as gender^12^, age^13^, hours of sleep^14^, physical activity and screentime^15^, family size^16^, life satisfaction^17^, residence area^18^, socioeconomic status^19^, self-rated health^20^, body mass index^21^, eating breakfast^22^, body image^23^, number of close friends^24^, having weight-reduction plan^25^, junk-food consumption^26^, sweetened beverage consumption^27^, smoking^28^, abdominal obesity^29^, parents’ education^30^, birth weight^31^, breastfeeding^32^, family history of sudden death^33^ and family history of cancer^34^. However, many studies focused on the univariate analysis and descriptive studies of psychological distress in children and adolescents, but there exist fewer studies about the classification of comprehensive modifiable risk factors and their interactions and associations with psychological distress.

Few studies have been conducted on using data mining for psychological distress classification, and to the best of our knowledge, none of them comprehensively considered various determinants and their interactions^3, 35, 36^. Thus, this study aims to classify the risk factors associated with psychiatric symptoms based on the demographic, lifestyle, socioeconomic status, and family history of diseases in a large sample of children and adolescents. The Group Method of Data Handling (GMDH), proposed by Ivakhnenko^37^, was used for classification in our study. In this network, optimal hyperparameters, the number of hidden layers, and neurons in such layers are automatically identified. Moreover, the interpretable interaction network is provided by the GMDH, which is very important in medical data mining^38^.

## Results

### Prevalence of mild-to-moderate emotional symptoms, worriedness, and depression

**Figure 1:**
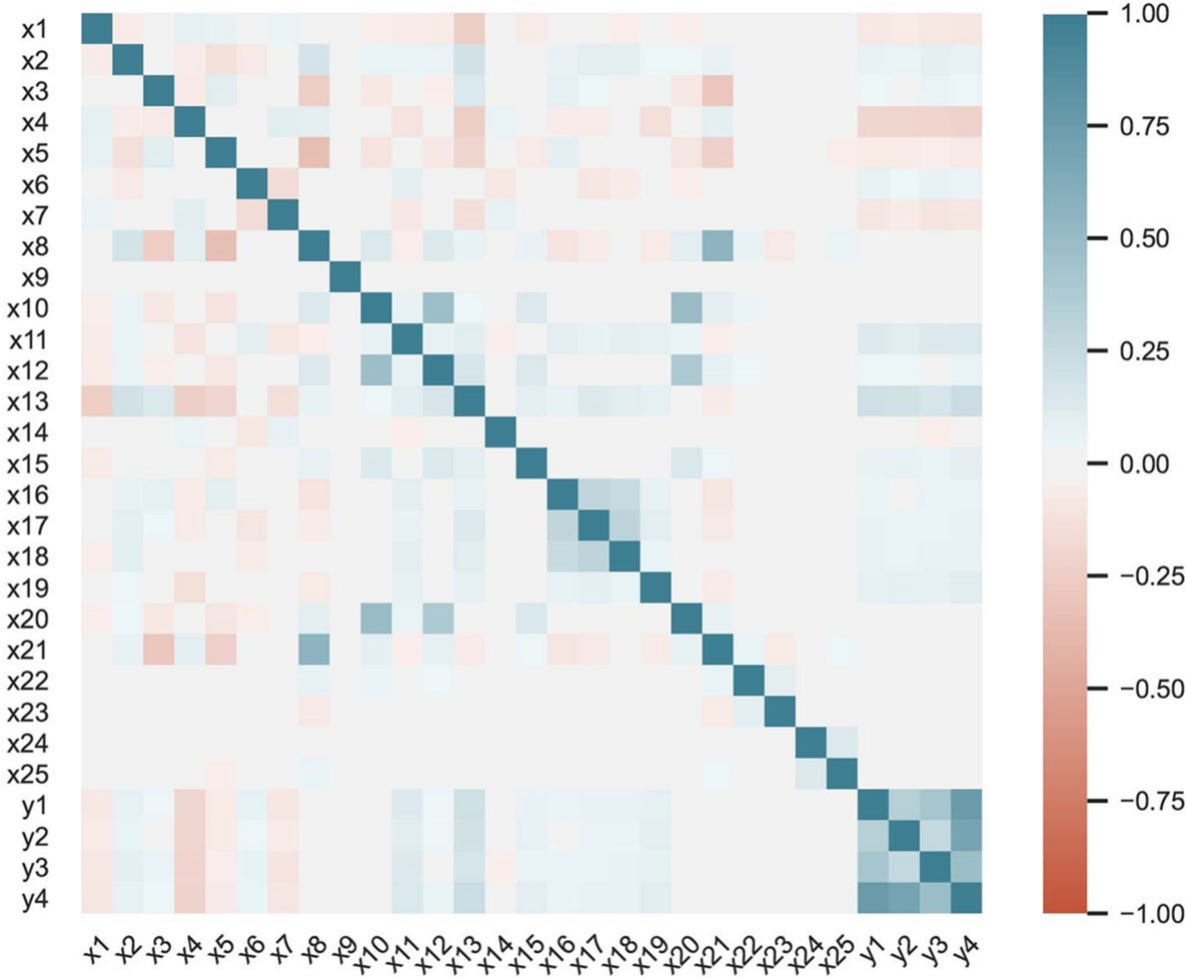
The bivariate correlation between the inputs and outputs. × 1: sleeping-time cat. (category); × 2: screen time cat.; × 3: family-size cat.; × 4: life satisfaction cat.; × 5: residence area; × 6: gender; × 7: physical activity cat.; × 8: SES cat.; × 9: self-rated health cat. (SRH); × 10: BMI cat.; × 11: breakfast cat.; × 12: body image cat.; × 13: age cat.; × 14: the number of close friends cat.; × 15: weight-reduction plan; × 16: salty-snack cat.; × 17: sweetened beverage consumption cat.; × 18: fast food consumption cat.; × 19: smoker; × 20: abdominal obesity; × 21: mother education cat.; × 22: birth-weight cat.; × 23: milk type during infancy; × 24: the family history of sudden death; × 25: the family history of cancer; y1: worriedness symptom; y2: depression symptom; y3: mild-to-moderate emotional symptoms; y4: psychiatric symptoms. The entire ordinal variables were encoded ascending (e.g., mild to severe, or seldom to daily), except for × 9 (1: good, 2: moderate, 3: bad), × 11 (1: non-skipper, 2: semi-skipper, 3: skipper), and × 17 (1: daily, 2: weekly, 3: seldom/never).

This national survey's participation rate was 90.6%, and the subjects enrolled were 13,486 children and adolescents out of 14,880 invited subjects. The number of missing values ranged from zero (living place and gender) to 1158 (birth weight category) in the enrolled subjects. The occurrence of missing data on the dependent and independent variables of the enrolled subjects was random (Little's MCAR test^39^; *p* = 0.421). In our study, the subjects with complete information were first analyzed. Accordingly, 10,350 subjects (i.e., 76.7% of the enrolled subjects) were analyzed. Overall, the percentages of 6–10, 11–14- and 15–19-year-old age groups were 33.7, 35.0, and 31.3, respectively, and 50.1% of the population was boys. The prevalence of having worriedness symptoms, mild-to-moderate emotional symptoms, and depression symptoms was 23.7%, 11.1%, and 20.1%, respectively. Specifically, the prevalence of having a worriedness symptom was 20.6% and 26.8% in boys and girls, respectively. 8.9% of boys and 13.2% of the girls suffered from mild-to-moderate emotional symptoms, while 18.5% of boys and 21.7% of girls experienced depression symptoms. The distribution of demographic variables, family history of diseases, and lifestyle factors was presented in different psychiatric groups (Tables 1, 2). The pairwise association between the input features and the outcome variables was shown in Fig. 1.

**Table 1:** Demographic and socioeconomic characteristics of participants: the CASPIAN-IV study.

Feature	Worriedness symptom		*p*	Depression symptom		*p*	Mild-to-moderate emotional problems		*p*
	No N (%)	Yes N (%)		No N (%)	Yes N (%)		No N (%)	Yes N (%)	
**Family size**									
Less/= 4	4035 (39.0)	1125 (10.9)	< 0.001	4166 (40.2)	994 (9.6)	0.035	4667 (45.1)	493 (4.8)	< 0.001
> 4	3861 (37.3)	1329 (12.8)		4104 (39.7)	1086 (10.5)		4538 (43.8)	652 (6.3)	
**Place living**									
Urban	5888 (56.9)	1985 (19.2)	< 0.001	6168 (59.6)	1705 (16.5)	< 0.001	6946 (67.1)	927 (9.0)	< 0.001
Rural	2008 (19.4)	469 (4.5)		2102 (20.3)	375 (3.65)		2259 (21.8)	218 (2.1)	
**Gender**									
Boy	4112 (39.7)	1069 (10.3)	< 0.001	4224 (40.8)	957 (9.2)	< 0.001	4720 (45.6)	461 (4.5)	< 0.001
Girl	3784 (36.6)	1385 (13.4)		4046 (39.1)	1123 (10.9)		4485 (43.3)	684 (6.6)	
**Socioeconomic status**									
Weak	2440 (23.6)	800 (7.7)	0.179	2584 (25.0)	656 (6.3)	0.948	2833 (27.4)	407 (4.0)	0.005
Moderate	2647 (25.6)	825 (8.0)		2780 (26.8)	692 (6.7)		3109 (30.0)	363 (3.5)	
Good	2809 (27.1)	829 (8.0)		2906 (28.1)	732 (7.1)		3263 (31.5)	375 (3.6)	
**BMI**									
Underweight	978 (9.5)	270 (2.6)	0.050	993 (9.6)	255 (2.5)	0.041	1108 (10.7)	140 (1.4)	0.980
Healthy weight	5239 (50.6)	1617 (15.6)		5523 (53.4)	1333 (12.9)		6098 (58.9)	758 (7.3)	
Overweight and obese	1679 (16.2)	567 (5.5)		1754 (16.9)	492 (4.7)		1999 (19.3)	247 (2.4)	
**Age categories**									
6–10	3071 (29.7)	416 (4.0)	< 0.001	3111 (30.1)	376 (3.6)	< 0.001	3342 (32.3)	145 (1.4)	< 0.001
11–14	2711 (26.2)	916 (8.9)		2898 (28.0)	729 (7.1)		3208 (31.0)	419 (4.0)	
15–19	2114 (20.4)	1122 (10.8)		2261 (21.8)	975 (9.4)		2655 (25.7)	581 (5.6)	
**Abdominal obesity: based on waist to height ratio**									
No	6396 (61.8)	1942 (18.8)	0.042	6703 (64.8)	1635 (15.8)	0.013	7426 (71.7)	912 (8.8)	0.412
Yes	1500 (14.5)	512 (4.9)		1567 (15.1)	445 (4.3)		1779 (17.2)	233 (2.3)	
**Mother education**									
Illiterate	1120 (10.8)	401 (3.9)	< 0.001	1202 (11.6)	319 (3.1)	0.064	1316 (12.7)	205 (2.0)	0.001
Diploma	6013 (58.1)	1870 (18.1)		6286 (60.7)	1597 (15.4)		7026 (67.9)	857 (8.3)	
University	763 (7.4)	183 (1.7)		782 (7.6)	164 (1.6)		863 (8.3)	83 (0.8)	
**Birth weight category**									
Less than 2500 g	705 (6.8)	251 (2.4)	0.042	755 (7.3)	201 (1.9)	0.423	831 (8.0)	125 (1.2)	0.109
2500–4000 g	6624 (64.0)	2000 (19.3)		6915 (66.8)	1709 (16.5)		7696 (74.4)	928 (9.0)	
More than 4000 g	567 (5.5)	203 (2.0)		600 (5.8)	170 (1.7)		678 (6.5)	92 (0.9)	
**Milk type during infancy**									
Others	1226 (11.8)	389 (3.8)	0.699	1297 (12.5)	318 (3.1)	0.657	1430 (13.8)	185 (1.8)	0.586
Mother milk	6670 (64.4)	2065 (20.0)		6973 (67.4)	1762 (17.0)		7775 (75.1)	960 (9.3)	
**Family history of sudden death**									
No	6940 (67.1)	2122 (20.5)	0.065	7271 (70.2)	1791 (17.3)	0.027	8084 (78.1)	978 (9.5)	0.023
Yes	956 (9.2)	332 (3.2)		999 (9.7)	289 (2.8)		1121 (10.8)	167 (1.6)	
**Family history of cancer**									
No	6727 (65.0)	2082 (20.1)	0.668	7081 (68.4)	1728 (16.7)	0.004	7848 (75.8)	961 (9.3)	0.238
Yes	1169 (11.3)	372 (3.6)		1189 (11.5)	352 (3.4)		1357 (13.1)	184 (1.8)	

*N* number of people who are in each category.

**Table 2:** Lifestyle and health-related characteristics of participants: the CASPIAN-IV study.

Feature	Worriedness symptom		*p*	Depression symptom		*p*	Mild-to-moderate emotional problems		*p*
	No N (%)	Yes N (%)		No N (%)	Yes N (%)		No N (%)	Yes N (%)	
**Sleeping time**									
< 5 h	25 (0.2)	27 (0.3)	< 0.001	32 (0.3)	20 (0.2)	< 0.001	37 (0.4)	15 (0.1)	< 0.001
5–8 h	1629 (15.8)	678 (6.5)		1736 (16.8)	571 (5.5)		1953 (18.9)	354 (3.4)	
> 8 h	6242 (60.3)	1749 (16.9)		6502 (62.8)	1489 (14.4)		7215 (69.7)	776 (7.5)	
**Screen time**									
<= 4	6600 (63.8)	1879 (18.1)	< 0.001	6865 (66.3)	1614 (15.6)	< 0.001	7653 (73.9)	826 (8.0)	< 0.001
> 4	1296 (12.5)	575 (5.6)		1405 (13.6)	466 (4.5)		1552 (15.0)	319 (3.1)	
**Physical activity**									
Mild	2408 (23.3)	986 (9.5)	< 0.001	2599 (25.1)	795 (7.7)	< 0.001	2858 (27.6)	536 (5.2)	< 0.001
Moderate	3026 (29.2)	860 (8.3)		3131 (30.3)	755 (7.3)		3500 (33.8)	386 (3.7)	
Severe	2462 (23.8)	608 (5.9)		2540 (24.5)	530 (5.1)		2847 (27.5)	223 (2.2)	
**Self-rated health**									
Good	6299 (60.9)	1800 (74%)	0.534	6612 (63.9)	1200 (57%)	0.139	7364 (71.1)	704 (61%)	0.644
Moderate	1481 (14.3)	303 (12%)		1523 (14.7)	401 (19%)		1703 (16.5)	200 (17%)	
Bad	116 (1.1)	351 (14%)		135 (1.3)	479 (24%)		138 (1.3)	241 (22%)	
**Breakfast categories**									
Non skipper	5719 (55.2)	1427 (13.8)	< 0.001	5900 (57.0)	1246 (12.0)	< 0.001	6523 (63.0)	623 (6.0)	< 0.001
Semi skipper	939 (9.1)	402 (3.9)		1051 (10.2)	290 (2.8)		1172 (11.4)	169 (1.6)	
Skipper	1238 (12.0)	625 (6.0)		1319 (12.7)	544 (5.3)		1510 (14.6)	353 (3.4)	
**Body image**									
Under weight	2679 (25.9)	800 (7.7)	< 0.001	2817 (27.2)	662 (6.4)	< 0.001	3088 (29.8)	391 (3.8)	< 0.001
Normal	3817 (36.9)	1086 (10.5)		3978 (38.4)	925 (8.9)		4443 (42.9)	460 (4.5)	
Overweight	1400 (13.5)	568 (5.5)		1475 (14.3)	493 (4.8)		1674 (16.2)	294 (2.8)	
**Number of close friend's category**									
Nothing	155 (1.5)	67 (0.6)	0.003	169 (1.6)	53 (0.5)	0.070	189 (1.8)	33 (0.3)	< 0.001
One	1096 (10.6)	397 (3.8)		1181 (11.4)	312 (3.0)		1299 (12.6)	194 (1.9)	
Two	1878 (18.2)	581 (5.6)		2005 (19.4)	454 (4.4)		2156 (20.8)	303 (2.9)	
Three or more	4767 (46.1)	1409 (13.6)		4915 (47.5)	1261 (12.2)		5561 (53.7)	615 (6.0)	
**Having nutrition plan based on special diet**									
No	6999 (67.6)	2046 (19.8)	< 0.001	7326 (70.8)	1719 (16.6)	< 0.001	8101 (78.3)	944 (9.1)	< 0.001
Yes	897 (8.7)	408 (3.9)		944 (9.1)	361 (3.5)		1104 (10.7)	201 (1.9)	
**Salty snack including chips**									
Seldom/never	4118 (39.8)	1183 (11.4)	< 0.001	4276 (41.3)	1025 (9.9)	< 0.001	4788 (46.2)	513 (5.0)	< 0.001
Weekly	2903 (28.0)	902 (8.7)		3062 (29.6)	743 (7.2)		3375 (32.6)	430 (4.1)	
Daily	875 (8.5)	369 (3.6)		932 (9.0)	312 (3.0)		1042 (10.1)	202 (2.0)	
**Sweetened beverage consumption**									
Seldom/never	4981 (48.1)	257 (2.5)	< 0.001	5184 (50.1)	223 (2.2)	< 0.001	6860 (66.3)	130 (1.3)	< 0.001
Weekly	2436 (23.6)	787 (7.6)		2573 (24.8)	650 (6.3)		2851 (27.5)	372 (3.6)	
Daily	479 (4.6)	1410 (13.6)		513 (5.0)	1207 (11.6)		5748 (55.5)	643 (6.2)	
**Fast food consumption**									
Seldom/never	5915 (57.2)	1698 (16.4)	< 0.001	6161 (59.5)	1452 (14.0)	< 0.001	606 (5.9)	753 (7.3)	< 0.001
Weekly	1802 (17.4)	652 (6.3)		1915 (18.5)	539 (5.2)		2124 (20.5)	330 (3.2)	
Daily	179 (1.7)	104 (1.0)		194 (1.9)	89 (0.9)		221 (2.1)	62 (0.6)	
**Passive or active smoker**									
No	4453 (43.0)	1139 (11.0)	< 0.001	4656 (45.0)	936 (9.0)	< 0.001	5111 (49.4)	481 (4.6)	< 0.001
Yes	3443 (33.3)	1315 (12.7)		3614 (34.9)	1144 (11.1)		4094 (39.6)	664 (6.4)	
**Life-satisfaction**									
Low	1241 (12.0)	1715 (16.6)	< 0.001	1313 (12.7)	1413 (13.7)	< 0.001	1537 (14.9)	702 (6.7)	< 0.001
High	6655 (64.3)	739 (7.1)		6957 (67.2)	667 (6.4)		7668 (74.1)	443 (4.3)	

*N* number of people who are in each category.

The association between outcome variables was measured using the Phi coefficient^40^. It was 0.208 (CI 95% 0.191–0.224) (*p* < 0.001) between worriedness and depression symptoms. The association between worriedness symptom and mild-to-moderate emotional symptomswas 0.385 (CI 95% 0.370–0.399) (*p* < 0.001). Similarly, the association between depression symptom and the mild-to-moderate emotional symptoms was 0.285 (CI 95% 0.269–0.300) (*p* < 0.001). Thus, no more than trivial association in the first and last outcome pairs was observed^40, 41^, while worriedness symptom and mild-to-moderate emotional symptoms were weakly correlated in our study.

Moreover, the association between depression symptoms and each of Questions 1–5 (i.e., questions used to define mild-to-moderate emotional symptoms) was assessed. The lowest association was with confusion (Q5) (Phi coefficient = 0.179 (CI 95% 0.162–0.195) (*p* < 0.001), while the highest association was with worthless (Q1) (Phi coefficient = 0.220 (CI 95% 0.203–0.236) (*p* < 0.001). Thus, no more than a trivial association between depression and any of the five mild-to-moderate emotional symptoms items was observed. The factor analysis was also used based on principal components analysis (PCA) on the original Q1–Q5. Only one PC was selected with Eigenvalues greater than one. It showed an acceptable discrimination for depression diagnosis [Area under the ROC Curve = 0.736 (CI 95% 0.726–0.746); (*p* < 0.001)]. Thus, the combination of Q1-Q5 could be indirectly used for depression symptom diagnosis.

### Classification results

**Figure 2:**
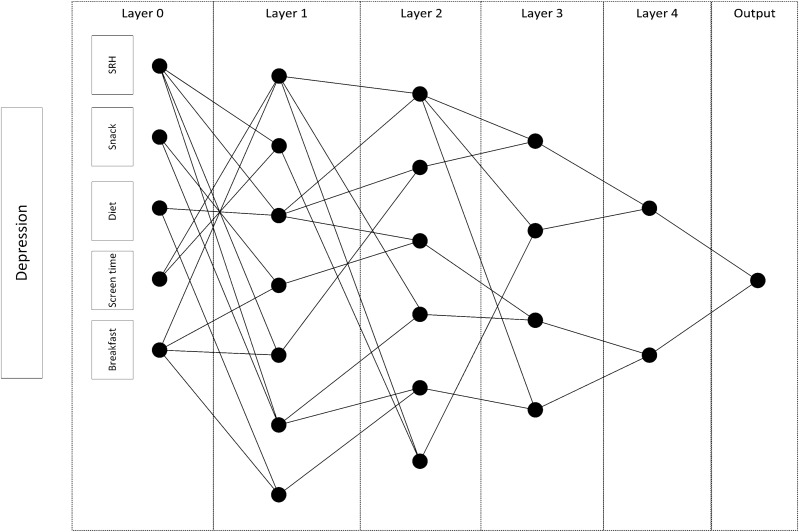
A representative GMDH network for classifying depression symptoms.

**Figure 3:**
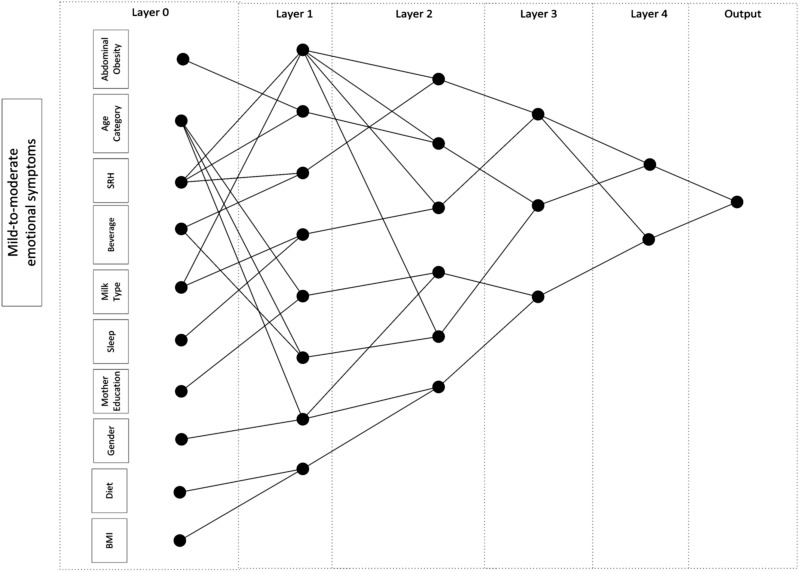
A representative GMDH network for classifying mild-to-moderate emotional symptoms.

**Figure 4:**
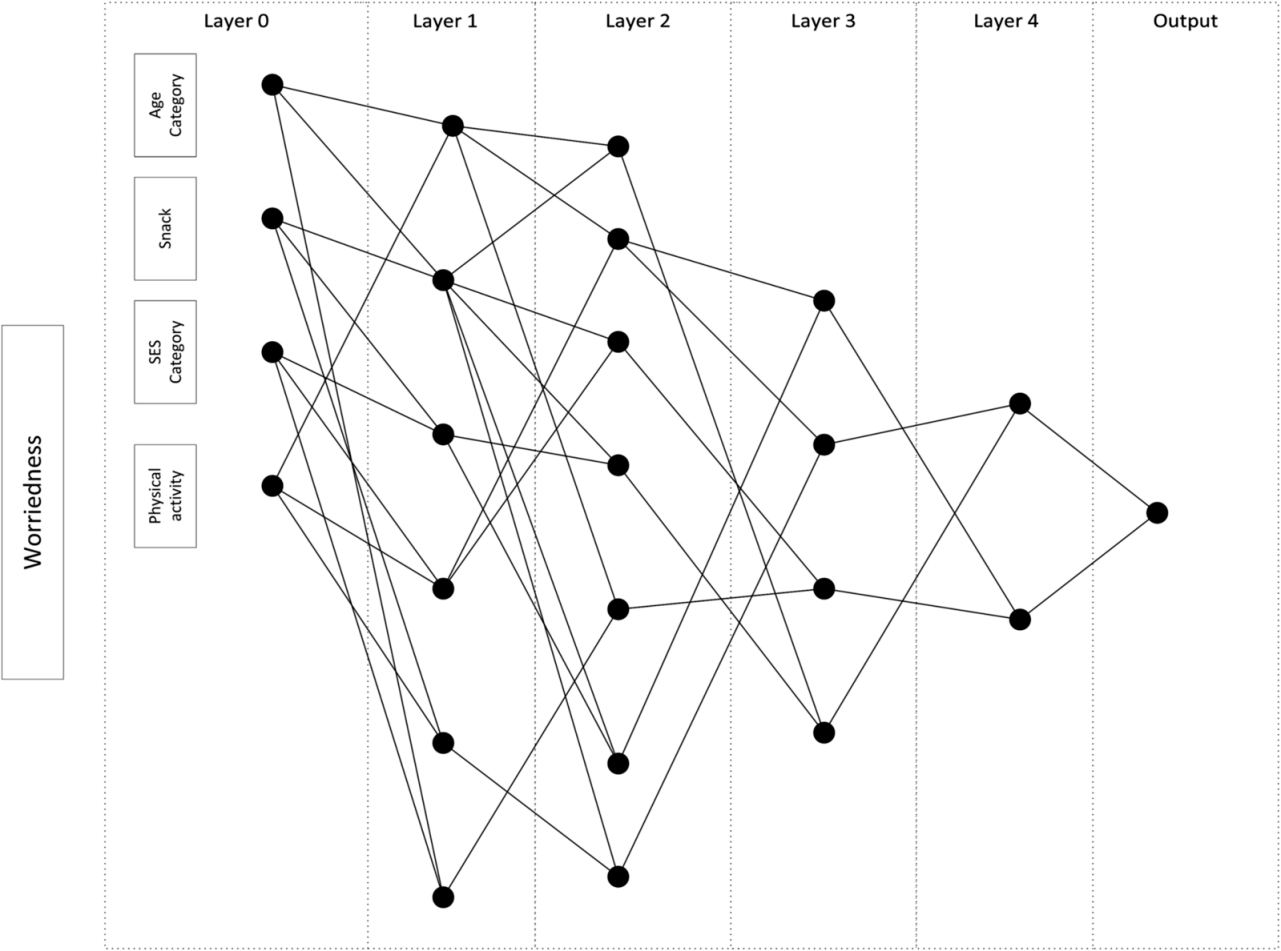
A representative GMDH network for classifying worriedness symptoms.

Among 25 features used in our study, five, ten, and four features were selected by the stability feature selection and the GMDH network for depression symptom, mild-to-moderate emotional symptoms, and worriedness symptom, respectively, consistent during cross-validation. The proposed GMDH networks for depression symptoms, mild-to-moderate emotional symptoms, and worriedness symptoms were shown in Figs. 2, 3 and 4. The top three most essential depression symptom features were the eating breakfast category (cat.), self-rated health cat (SRH), and diet program. They were age cat., self-rated health cat., and milk type for the mild-to-moderate emotional symptoms while age cat., physical activity cat., and socioeconomic status (SES) cat. were the most critical features for worriedness symptom. The most important features were selected based on the number of interactions used in the network.

The average performance of the GMDH network and the state-of-the-art on the test folds during threefold cross-validation of psychological distress were shown in Table 3. The entire performance indices and their CI 95% of the analyzed methods on the cross-validated confusion matrix were reported in Table 4. The GMDH network significantly outperformed the state-of-the-art for the entire outcomes (adjusted *p* < 0.05; McNemar's test), except for LDA in mild-to-moderate emotional symptoms classification where they were not significantly different.

**Table 3:** The performance of the different classifiers in MEAN ± SD over the test folds using threefold cross-validation.

Outcome	Classifier	Se (%)	Sp (%)	Pr (%)	Acc (%)
Depression symptom	GMDH	78 ± 3	96 ± 1	83 ± 4	92 ± 2
	LDA	62 ± 2	66 ± 1	31 ± 1	65 ± 1
	SVM	19 ± 2	91 ± 2	36 ± 5	77 ± 1
	MLP	10 ± 3	98 ± 1	57 ± 3	80 ± 1
Mild-to-moderate emotional symptoms	GMDH	70 ± 3	70 ± 2	23 ± 2	70 ± 2
	LDA	64 ± 3	70 ± 1	21 ± 1	70 ± 1
	SVM	14 ± 1	96 ± 1	29 ± 2	87 ± 1
	MLP	4 ± 4	99 ± 1	45 ± 11	89 ± 1
Worriedness symptom	GMDH	70 ± 3	88 ± 2	64 ± 3	84 ± 2
	LDA	62 ± 1	65 ± 1	35 ± 1	64 ± 1
	SVM	23 ± 3	88 ± 2	38 ± 1	73 ± 1
	MLP	13 ± 1	97 ± 1	54 ± 3	77 ± 1
Psychiatric symptoms	GMDH	80 ± 2	70 ± 3	59 ± 2	74 ± 2
	LDA	63 ± 1	66 ± 1	50 ± 1	65 ± 1
	SVM	32 ± 2	87 ± 3	57 ± 4	67 ± 2
	MLP	32 ± 1	88 ± 1	60 ± 2	69 ± 1

*Se* Sensitivity, *Sp* Specificity, *Pr* Precision, *Acc* Accuracy.

**Table 4:** The performance of the different classifiers and their CI 95% based on the cross-validated confusion matrix.

Outcome	Classifier	Se (%)	Sp (%)	Pr (%)	NPV (%)	Acc (%)	AUC	DOR	DP	MCC	K(C)
Depression symptom	GMDH	79 (77, 81)	97 (96, 97)	87 (85, 88)	95 (94, 95)	93 (93, 94)	0.88 (0.87, 0.89)	121.6 (103.2, 143.4)	2.04 (1.97, 2.11)	0.79 (0.78, 0.80)	0.79 (0.77, 0.80)
	LDA	62 (60, 64)	66 (65, 67)	31 (30, 33)	87 (87, 88)	65 (64, 66)	0.64 (0.63, 0.65)	3.2 (2.9, 3.5)	0.48 (0.44, 0.53)	0.23 (0.21, 0.25)	0.20 (0.18, 0.22)
	SVM	19 (17, 21)	91 (91, 92)	36 (33, 39)	82 (81, 83)	77 (76, 78)	0.55 (0.54, 0.57)	2.5 (2.2, 2.9)	0.39 (0.33, 0.45)	0.14 (0.12, 0.15)	0.13 (0.10, 0.16)
	MLP	10 (9, 12)	98 (97, 98)	57 (52, 62)	81 (81, 82)	80 (80, 81)	0.54 (0.53, 0.56)	5.9 (4.7, 7.2)	0.75 (0.66, 0.84)	0.18 (0.16, 0.20)	0.12 (0.09, 0.16)
Mild-to-moderate emotional symptoms	GMDH	71 (68, 74)	69 (68, 70)	22 (21, 24)	95 (94, 96)	69 (68, 70)	0.70 (0.68, 0.72)	5.5 (4.8, 6.2)	0.72 (0.66, 0.78)	0.26 (0.24, 0.28)	0.20 (0.18, 0.23)
	LDA	64 (62, 67)	70 (69, 71)	21 (20, 23)	94 (94, 95)	70 (69, 71)	67 (66, 69)	4.3 (3.7, 4.8)	0.62 (0.56, 0.67)	0.23 (0.21, 0.25)	0.18 (0.16, 0.21)
	SVM	14 (12, 16)	96 (95, 96)	29 (25, 33)	90 (89, 91)	87 (86, 87)	0.55 (0.53, 0.57)	3.7 (3.0, 4.5)	0.55 (0.47, 0.63)	0.13 (0.12, 0.15)	0.12 (0.08, 0.17)
	MLP	4 (3, 6)	99 (99–100)	50 (40, 60)	89 (89, 90)	89 (88, 89)	0.52 (0.51, 0.54)	8.4 (5.6, 12.4)	0.90 (0.73, 1.07)	0.12 (0.10, 0.14)	0.06 (0.01, 0.11)
Worriedness symptom	GMDH	71 (69, 73)	86 (85, 88)	61 (59, 63)	91 (90, 91)	82 (81, 83)	0.79 (0.77, 0.80)	15.0 (13.5, 16.8)	1.15 (1.10, 1.20)	0.54 (0.53, 0.56)	0.54 (0.52, 0.56)
	LDA	62 (60, 64)	65 (64, 66)	35 (34, 37)	84 (84, 85)	64 (63, 65)	0.63 (0.62, 0.65)	3.0 (2.7, 3.3)	0.46 (0.42, 0.50)	0.23 (0.21, 0.25)	0.21 (0.19, 0.23)
	SVM	23 (22, 25)	88 (87, 89)	38 (35, 40)	79 (78, 80)	73 (72, 74)	0.56 (0.54, 0.57)	2.2 (2.0, 2.5)	0.34 (0.29, 0.39)	0.14 (0.12, 0.16)	0.13 (0.10, 0.16)
	MLP	13 (12, 14)	97 (96, 97)	54 (50, 58)	78 (77, 79)	77 (76, 77)	0.55 (0.53, 0.56)	4.1 (3.5, 4.9)	0.60 (0.53, 0.67)	0.17 (0.16, 0.19)	0.13 (0.10, 0.16)
Psychiatric symptoms	GMDH	78 (77, 79)	71 (70, 72)	59 (58, 61)	86 (85, 87)	73 (72, 75)	0.75 (0.73, 0.76)	8.7 (7.9, 9.5)	0.92 (0.88, 0.96)	0.47 (0.45, 0.48)	0.46 (0.44, 0.47)
	LDA	63 (61, 64)	66 (65, 67)	50 (48, 51)	76 (75, 78)	65 (63, 66)	0.64 (0.63, 0.65)	3.2 (3.0, 3.5)	0.50 (0.46, 0.53)	0.27 (0.26, 0.29)	0.27 (0.25, 0.29)
	SVM	32 (30, 33)	87 (86, 87)	56 (54, 58)	70 (69, 71)	67 (66, 68)	0.59 (0.58, 0.60)	3.0 (2.7, 3.3)	0.47 (0.43, 0.51)	0.22 (0.20, 0.24)	0.20 (0.18, 0.23)
	MLP	33 (31, 34)	88 (87, 89)	60 (58, 62)	71 (70, 72)	69 (68, 70)	0.60 (0.59, 0.61)	3.6 (3.3, 4.0)	0.54 (0.50, 0.59)	0.25 (0.23, 0.27)	0.23 (0.21, 0.25)

*Se* sensitivity, *Sp* specificity, *Acc* accuracy, *Pr* precision, *F1S* F1-Score, *AUC* area under the receiver operating characteristic (ROC) curve, *LR* likelihood ratio, *DOR* diagnosis odds ratio, *MCC* Matthews correlation coefficient, *DP* discriminant power, *K(C)* Cohen's kappa coefficient, *NPV* negative predictive value. The entire AUC and K(C) values were statistically significant (*p* < 0.05). Statistical power (Power), false alarm (FA), and F1-score (F1S) values could be directly calculated as Power = Se, FA = 1 − Sp(%)/100, and F1S = 2 * Se * Pr/(Se + Pr).

We further classified the imputed dataset in which the number of subjects was increased from 10,350 to 11,820 using the GMDH algorithm (Table 5). No significant improvement was observed compared with the dataset with complete information (adjusted *p* > 0.05; McNemar's test).

**Table 5:** The performance of the GMDH classifier in MEAN ± SD over the test folds using threefold cross-validation on the imputed dataset.

Outcome	Se (%)	Sp (%)	Pr (%)	Acc (%)
Depression symptom	79 ± 4	95 ± 2	82 ± 3	91 ± 3
Mild-to-moderate emotional symptoms	71 ± 2	71 ± 3	40 ± 3	71 ± 1
Worriedness symptom	72 ± 2	87 ± 3	41 ± 2	85 ± 1
Psychiatric symptoms	81 ± 3	71 ± 2	81 ± 2	77 ± 3

*Se* Sensitivity, *Sp* Specificity, *Pr* Precision, *Acc* Accuracy.

The performance of the proposed algorithm for classifying extended three-class psychiatric symptoms was provided in Table 6. The algorithm was run for each age category (6–10, 11–14, and 15–19 years) to improve its performance. The selected factors by the stability feature selection were breakfast, life satisfaction, self-rated health, screen time, residence area, sleeping-time, and weight-reduction plan for the first age category. They were self-rated health, life satisfaction, gender, breakfast, sleeping-time, residence area, weight-reduction plan, physical activity, and body image for the second age category. The algorithm selected self-rated health, life satisfaction, gender, breakfast, physical activity, body image, and screen time for the third age category. Among the selected factors, self-rated health, life satisfaction, and breakfast were common in all age categories.

**Table 6:** The performance of the GMDH algorithm (in percent) based on the cross-validated confusion matrix for classifying extended three-class psychiatric symptoms.

Age category (year)	Low-risk		Medium-risk		High-risk		Overall				
	Pr	Se	Pr	Se	Pr	Se	Pr_M_	Se_M_	F_1_S_M_	Acc	AR (CI 95%)
6–10	73	47	70	56	73	88	72	64	68	72	50 (47–52)
11–14	72	68	66	66	65	70	68	68	68	68	52 (50–54)
15–19	75	81	68	67	69	57	71	68	70	72	54 (52–56)

*Pr* Precision, *Se* Sensitivity, *Acc* Accuracy, *Pr*_*M*_ Marco-averaged precision, *Se*_*M*_ Marco-averaged recall (= sensitivity), *F*_*1*_*S*_*M*_ Marco-averaged F_1_ Score, *AR* Agreement rate (Cohen’s Kappa), *CI* confidence interval.

## Discussion

In our study, we considered depression symptoms, worriedness symptoms, and mild-to-moderate emotional symptoms for classification. The SRH was recognized as the primary variable for classifying depressive symptoms and mild-to-moderate emotional symptoms (Figs. 2, 3). Overall, few studies were performed on SRH in the literature. It has been shown that depression was strongly associated with reporting poor SRH^42^. There was also a relationship between poor SRH, depressive and anxiety symptoms among university students with high academic stress^43^.

Moreover, a longitudinal study of adolescent health in the United States demonstrated that one of the main factors associated with persistent depressive symptoms was poor self-rated general health^44^. Although there was no significant correlation between SRH and depression symptoms in our dataset (Rank Biserial r_rb_ = 0.002; *p* = 0.819) (Fig. 1), its interactions with screentime, diet, and breakfast were selected by the GMDH network (Fig. 2). It is how the interaction network identifies indirect factors. However, the univariate analysis could not identify this factor. It was similar for mild-to-moderate emotional symptoms, where there was no significant correlation between SRH and mild-to-moderate emotional symptoms in our dataset (Rank Biserial r_rb_ = 0.008; *p* = 0.390) (Fig. 1); its interactions with milk type, abdominal obesity, and beverage consumption were selected by the GMDH network (Fig. 3).

One of the most critical risk factors for children, the physical activity level, was selected for those having worriedness symptoms in our study (Fig. 4). The beneficial effects of regular physical activity on health are indisputable in modern medicine^45^. Furthermore, a large amount of exercise plays an essential role in minimizing the worry in clinical settings^46^. Although the correlation between physical activity and worriedness symptoms was very low in our study (Rank Biserial r_rb_ = −0.087; *p* =  <0.001) (Fig. 1), its interactions with the others were selected by the GMDH network (Fig. 4).

In our study, sleep hour was selected as a factor for mild-to-moderate emotional symptoms (Fig. 3). It is in agreement with previous studies^47^. Adverse general health outcomes are associated with the indicators of sleep problems, such as short sleep duration. Another study on 11,788 pupils from 11 different European countries showed a negative association between sleep time hours per night and emotional symptoms^48^. Although the correlation between sleep hours and mild-to-moderate emotional symptoms was very low in our study (Rank Biserial r_rb_ = −0.080; *p* =  <0.001) (Fig. 1), its interaction with the milk type during infancy was selected by the GMDH network (Fig. 4). It was shown in the literature that there is a relationship between breastfeeding and sleep quality in infants^49^. Also, breastfeeding is related to behavior problems in children and adolescents^50^. However, there was no significant correlation between sleep hour and milk type in our study (Rank Biserial r_rb_ = 0.005; *p* = 0.634) (Fig. 1).

One of the notable factors in our research was screen time (Fig. 2). Some studies showed that children who watch TV for more than two hours a day usually have lower self-esteem, lower school performance, and unhealthy eating habits^51^. Such consequences would lead to psychological distress in young children^52^. The consequences reported by these articles are in agreement with our findings of the positive association between screen time and having depression symptoms. Although the correlation between screen time and depression ssymptoms was very low in our study (Rank Biserial r_rb_ = 0.056; *p* =  < 0.001) (Fig. 1), its interactions with SRH and breakfast were selected by the GMDH network (Fig. 4).

A predictor of depression and worriedness symptoms was salty snack consumption (Figs. 2, 4). In general, there were few studies in this field^26^. It is also demonstrated that 12 to 13-year-old Norwegian adolescents with healthy dietary patterns have better mental health conditions^53^. Although the correlation between salty snack consumption and depression or worriedness symptoms was very low in our study (depression: Rank Biserial r_rb_ = 0.030; *p* = 0.002, worriedness: Rank Biserial r_rb_ = 0.044; *p* < 0.001) (Fig. 1), its interactions with SRH and breakfast, and with age category, SES category, and the physical activity were selected for depression, and worriedness symptoms, respectively (Figs. 2, 4).

Breakfast is one of the most important meals. The prevalence of breakfast skipping is increasing among adolescents. Previous studies showed that breakfast intake is related to mental problems^54^. Another study showed that skipping breakfast at least four times a week was significantly associated with a higher depressed mood score^55^. Our finding is consistent with such results on the association of breakfast consumption with depression symptoms (Fig. 2). Although the correlation between breakfast consumption and depression symptoms was relatively low in our study (Rank Biserial r_rb_ = 0.107; *p* < 0.001) (Fig. 1), its interactions with the others were selected (Fig. 2).

Discretization was used in our study to generate categorical input variables instead of interval variables. Although this procedure reduces the flexibility of the variables, it could increase the classifiers' performance and their generalization. We further used the original interval variables, and the average accuracy of the GMDH classification system was reduced by 3%, 4%, 2%, and 7% for depression symptoms, mild-to-moderate emotional symptoms, worriedness symptoms, and psychiatric symptoms. Moreover, the correlation between the original input variables and their categorical version ranged from 0.68 for age (Spearman’s rho; *p* < 0.001) to 0.95 for screentime variables (Spearman’s rho; *p* < 0.001). Thus, such a discretization did not significantly reduce the amount of information.

In our study, the GMDH network was used as a classifier. This network is incremental and expands with regularized least squares (RLS), a convex algorithm, but it also generates the interaction network (Figs. 2, 3, 4), leading to better clinical interpretations. The proposed GMDH network had very good diagnostic accuracy for depression symptom classification, while it showed good diagnostic accuracy for other outcomes. It showed an excellent, fair to good agreement rate with the gold standard for depression symptoms and worriedness symptom or psychiatric symptoms classification. However, it showed a poor agreement rate for the mild-to-moderate emotional symptoms classification. The proposed system's discriminant power was fair, limited for depression symptoms and worriedness symptom classification. However, it was poor for mild-to-moderate emotional symptoms classifications. The proposed system's false alarm (FA) ranged from 3 to 31% when classifying depression symptoms and mild-to-moderate emotional symptoms. However, the proposed system's statistical power was always higher than 70% in the entire outcome. Moreover, the false discovery rate (a.k.a., 1-precision) ranged from 13 to 78% for classifying depression symptoms and mild-to-moderate emotional symptoms.

The proposed GMDH network had the best and worst classification performance (MCC) for depression symptoms and mild-to-moderate emotional symptoms, respectively (Table 4). The dataset was highly imbalanced for mild-to-moderate emotional symptoms outcome (the prevalence of 11.1%). However, the entire performance indices were consistent in different test folds (Table 3). It must be mentioned that the diagnostic accuracy of classifying psychological distress is not usually high in the literature^56^. In the meanwhile, there could be two reasons why the GMDH significantly outperformed the MLP classifier. First, the MLP is a fully connected network, while the GMDH is not (Figs. 2, 3, 4), resulting in more parameters in the MLP. Second, the cost function of the GMDH was customized for the imbalanced data, while the cross-entropy was used for the MLP that could be improved by using weighted cross-entropy^57^.

The GMDH algorithm was further used to classify the extended three-class psychiatric symptoms. Overall, the agreement rate of the extended system was comparable to that of the four two-class problems (Tables 4, 6). However, the original questions are used in the extended system, and the severity of the entire psychiatric symptoms are identified rather than defining a psychiatric symptom based on one question. Accordingly, it might be preferred in practice. For the extended system, analyzing the interaction network identified that self-rated health, life satisfaction, breakfast consumption, and sleeping/screen time were the most critical factors.

In our study, we designed classification systems for psychiatric symptoms. Proper diagnosis of mental disorders, such as depressive and anxiety disorders, requires detailed analysis^58^. As an important limitation of our study, each outcome variable of depression and worriedness symptoms was derived from a single question. Mild-to-moderate emotional symptoms outcome was created based on five questions (confusion, insomnia, anxiety, angriness, and worthlessness). Generally, depression might be embedded in emotional symptoms, as one of the limitations of our methodology. However, due to the definition of depression in our study that prevented students from routine activities, it was more alarming than mild-to-moderate emotional symptoms defined based on Q1–Q5. However, Q1–Q5 could be considered as symptoms of depression. In our analysis, no more than a trivial association between depression and any of the five mild-to-moderate emotional symptoms items was observed. However, the combination of Q1–Q5 had acceptable discrimination for depression diagnosis. Moreover, the overall outcome (i.e., psychiatric symptoms) was generated based on the entire seven questions in our analysis.

The advantage of our study is the large sample size. Moreover, it analyzed the comprehensive factors related to psychiatric symptoms to monitor direct and indirect modifiable factors. However, it is a repeated cross-sectional study^59^, and no casualty can be inferred. We only analyzed the CASPIAN-IV data, and examining the trend and association between variables over time is the focus of our future activity. The other limitation was the possible bias in the self-reported answers of participants. Moreover, there is evidence about adolescents using the substance in Iran and their considerable psychological dysfunction^60^. However, it was not recorded in CASPIAN-IV. It is another limitation of our study.

In conclusion, our study emphasized the modifiable factors of psychiatric symptoms, including breakfast, salty snack, sweet beverage consumption, consumption, screentime, (abdominal) obesity, sleep hour, and physical activity. Iran ranked fourth among the countries with the highest age-standardized mental disorder DALYs rates (2436.44 DALYs per 100,000, based on the GBD 2019)^61^. Such disorders could root from childhood psychiatric symptoms, and empowering protective factors and changing modifiable risk factors might reduce such rates in the future. It is possible to design an online web-based or Android App of the developed algorithm to identify whether the student could have a high risk of “psychiatric symptoms” based on the selected input variables (Figs. 2, 3, 4). Such indirect health screenings have a great potential to be integrated into schools, which is the focus of our future activities.

## Methods

A large population of the fourth study of a national surveillance program, entitled “Childhood and Adolescence Surveillance and Prevention of Adult Non-communicable disease” (CASPIAN-IV), supported by the WHO/Eastern Mediterranean region and the Iranian Ministry of Health and the Ministry of Education, were analyzed in our project. Detailed methodology is published elsewhere^62^. We briefly describe the study population.

### The population and sampling method

A sample of 14,880 students aged 6–18 years was selected by multi-stage sampling from schools of urban and rural areas of 30 Iranian provinces. Having explained the objectives and protocols, participants were enrolled in the study. Parents gave the written informed consent and oral permission, while oral assent was obtained from students to express willingness to participate in research. Trained healthcare professionals performed all the data collection procedures. Study protocols were reviewed and approved by the Research and Ethics Council of Isfahan University of Medical Sciences (#5429-90). All procedures performed in studies involving human participants were in accordance with the ethical standards of the institutional and/or national research committee and with the 1964 Helsinki Declaration and its later amendments or comparable ethical standards.

### Outcome variables

The World Health Organization-Global School-based Student Health Survey (WHO-GSHS) was used in our study. It covers alcohol, tobacco use, hygiene, physical activity, mental health, dietary behaviors, violence, protective factors, and unintentional injuries among children and youths. After translating questions into Persian and simplifying the questions with any difficulty in understanding, the questionnaire's reliability and validity were assessed^63–65^. We considered psychiatric symptoms as depression symptoms, worriedness symptoms, and mild-to-moderate emotional symptoms, where the latter included confusion, insomnia, anxiety, angriness, and worthlessness^66^. The three indicated psychiatric symptoms were taken into consideration in this study. Also, if a subject has any of the depression symptoms, worriedness symptoms, and mild-to-moderate emotional symptoms, he/she was considered having “psychiatric symptoms” as the overall outcome of the study. The psychiatric symptoms were assessed by the questions presented in Supplementary Table S1. The first five questions were used to identify mild-to-moderate emotional symptoms in our research. Those who experienced at least 3 out of 5 problems every day, more than once a week, or once a week were defined as “adolescents with mild-to-moderate emotional symptoms.” An indicator of depression symptoms was a positive answer to the 6th question. In the last question, students who were worried most of the time or always so that they could not sleep at night were considered having worriedness symptoms^35^. Thus, our goal is to design and implement four binary classifiers to classify depression symptoms, mild-to-moderate emotional symptoms, worriedness symptoms, and “psychiatric symptoms” using the input variables. We further created an extended outcome using the entire seven questions used in the questionnaire. The original response to seven questions of the questionnaire was analyzed, and no dichotomization was used. The first principal component was extracted using Principal Component Analysis (PCA). Then, tertiles of the PC were extracted, splitting the subjects into “low,” “medium,” and “high” risk psychiatric symptoms groups. Thus, an extended three-class classification problem was also considered.

### Input variables

The input variables considered in our psychiatric symptom classification system are as follows^67^: age category, sex, socioeconomic status (SES), physical activity level, body mass index (BMI) category, abdominal obesity, family size, residence area, sleep-time category, screen time category, (passive and active) smoking habit, life satisfaction, health status, as well as the consumption of breakfast, fast food, salty snack and beverage, having nutrition plan, the number of close friends, mothers' education level, body image, birth weight category, milk type used in infancy, and the family history of cancer and sudden death.

### Measurements

In this study, age was categorized as 6–10, 11–14, and 15–19 years^68^. The screen time was considered as a categorical variable and consisted of the time spent on watching television (TV)/video and computer games during leisure time, less than or equal to 4 (≤ 4 h) defined as low, and greater than 4 (> 4 h), as high^35^. The three categories of sleep time hour per week were defined as: sleep time hour less than or equal to 5 (h ≤ 5 h) (low), 5–8 h (moderate), and ≥ 8 h (high)^69^. The residence area was considered either urban or rural. The physical activity at school and out of school was quantified using principal component analysis (PCA). The obtained scores were then categorized into tertiles^66^. Variables including family assets, such as ownership of a house, car, computer, occupation, and education level of parents, school type (private/public), were summarized in one main PCA component. Students were then classified as having low, moderate, and high SES, based on the component tertiles. The active smoking habit was considered using tobacco products (cigarettes, pipe, hookah, etc.) every day, while passive smoking was considered, as exposure to tobacco smoke was used by others or second-hand smokers^66^. Subjects with either passive or active smoking were considered as smokers and non-smokers; otherwise. The general state of participant's health was determined by the self-rated health (SRH) variable, asking “How would you describe your general state of health?” on the GSHS questionnaire, with the categories of “good,” “moderate,” and “bad”^35^. Life satisfaction was evaluated by asking questions about the degree of satisfaction with their life, using a tenth-point scale from 10 = very satisfied to 1 = very dissatisfied. The scores below 6, was signified low and high satisfaction, otherwise^70^. Body image was assessed using the question, “What do you think regarding your body size?”; the answer to this question was obtained with the following options “much too fat,” “a bit too fat,” “about the right size,” “a bit too thin,” “ much too thin.” For the analysis, the variable was divided into overweight (much too fat and a bit too fat), underweight (much too thin and a bit too thin), versus normal weight cognition^71^. Breakfast consumption was categorized into three groups as non-skipper (those eating breakfast 5–7 days a week, semi-skipper (those eating breakfast 3–4 days a week), and skipper (those eating breakfast 0–2 days a week)^22^. The students were asked about the frequency of salty snack consumption, categorized as “seldom or never,” “weekly,” and “daily” consumption^72^. The family size was categorized as “less than or equal to 4” or “greater than 4”. The number of close friends was categorized as nothing, one, two, three, or more. The nutrition plan was assessed as “adherence to a weight-modifying plan based on a special diet” or not, otherwise^71^. Sugar-sweetened beverage consumption (i.e., soda, soft drinks) was categorized as “daily,” “weekly,” “seldom or never”^72^. The consumption of fast foods (pizza, fried chicken, cheeseburgers, hamburgers, and hot dogs) was categorized into three groups: daily, Weekly, seldom, or never. The education level of mothers was categorized into three groups: Illiterate, diploma, and university degrees. Participants' birth weight (BW; g) was asked from their parents and then categorized into three groups; low (BW < 2500 g), normal (BW: 2500–4000 g), and high (BW > 4000 g)^73^. We also assessed whether breastfeeding was done for the children and adolescents during their infancy^74^, and the variable milk type was categorized as breast milk (1) and others (0) otherwise. Moreover, we considered the family history of sudden death (yes or no) and also the family history of cancer (yes or no) of the first-degree relatives of the subjects enrolled in the study.

### Anthropometric measurement

In our study, trained healthcare providers performed anthropometric measurements at school. All measurements were conducted with calibrated instruments, according to standard protocols^66^. Height was measured in the standing position, barefooted while shoulders touch the wall. It was recorded to the nearest 0.2 cm. We measured weight shoeless and in lightly dressed condition to the nearest 200 g. Waist circumference (WC) was measured by a non-elastic tape to the nearest 0.2 cm.

We calculated the BMI as weight in kilograms, divided by height in meters squared (m^2^). The subjects were classified as underweight, healthy weight, overweight or obese, if BMI was < 5th percentile, between 5th and 85th percentiles, higher than 85th percentiles (i.e., BMI categories), respectively^75^. Abdominal obesity was defined as WC to height ratio (WHtR) of more than 0.5^76^.

### Feature extraction

The interval variables (e.g., age, BMI, birth weight, family size, number of close friends, sleep, and screen time) were first categorized using unsupervised discretization. Although discretization reduces interval variables' flexibility, it could improve the classification problems' performance and generalization^77^. The input features in our study were thus entirely categorical. Their measurement scale was nominal (e.g., sex, smoking status, milk type, family history of sudden death, family history of cancer) or ordinal (e.g., age category, SES, physical activity level, BMI category). For each categorical variable, the wight-of-evidence encoding^78^ was used for obtaining continuous covariates. For each outcome variable, stability feature selection^79^ was then performed. The selected features were used in the following classification procedure.

### Classification

Twenty-five input variables were used in GMDH (i.e., layer zero), while the outcomes depression, worriedness, and mild-to-moderate emotional symptomswere separately used as outputs. At the first layer, each pairwise interaction of the inputs was considered as a neuron. Suppose that the pair x_i,j,_ and x_i,k_ (features no. k and j from the subject no. i) are combined to generate the estimated outcome ${\tilde{y }}_{i}$ at the first layer using the second-order polynomial model shown in Eq. (1).

1 $${\tilde{y }}_{i}={a}_{0}+{a}_{1}\times {x}_{i,j}+{a}_{2}\times {x}_{i,k}+{a}_{3}\times {x}_{i,j}^{2}+{a}_{4}\times {x}_{i,k}^{2}+{a}_{5}\times {x}_{i,j}\times {x}_{i,k}$$

where the coefficients $A={\left[{a}_{0},\dots ,{a}_{5}\right]}^{T}$ could be estimated using Regularized Least Squares (RLS)^80^ on the entire estimation set in Eqs. (2,3).

2 $$A={\left({X}^{T}\times X+\lambda {I}_{6}\right)}^{-1}\times {X}^{T}\times Y$$

where

3 $${X}_{{N}_{e}\times 6}=\left[\begin{array}{cccccc}1& {x}_{1,j}& {x}_{1,k}& {x}_{1,j}^{2}& {x}_{1,k}^{2}& {x}_{1,j}\times {x}_{1,k}\\ 1& {x}_{2,j}& {x}_{2,k}& {x}_{2,j}^{2}& {x}_{2,k}^{2}& {x}_{2,j}\times {x}_{2,k}\\ .& .& .& .& .& .\\ .& .& .& .& .& .\\ .& .& .& .& .& .\\ 1& {x}_{{N}_{e},j}& {x}_{{N}_{e},k}& {x}_{{N}_{e},j}^{2}& {x}_{{N}_{e},k}^{2}& {x}_{{N}_{e},j}\times {x}_{{N}_{e},k}\end{array}\right],{Y}_{{N}_{e}\times 1}={\left[{y}_{1},{y}_{2},\dots ,{y}_{{N}_{e}}\right]}^{T}$$

and λ is the regularization parameter, y_i_ is the output of the sample no. i of the estimation set and I_6_ is the identity matrix of size six. In addition to the regularization, the training set was divided into the estimation set with N_e_ number of samples and the validation set with N_v_ number of samples to avoid over-fitting during learning. The regularization parameter was tuned using the brute-force search algorithm to maximize the Matthews correlation coefficient (MCC)^81^ on each output validation set.

At the first layer, each neuron's RLS coefficients were estimated on the estimation set using the above procedure. Each neuron's performance was then assessed on the validation set, and the corresponding MCC values were calculated. The top 10 neurons with better performance than the previous layer's neurons were selected at maximum, and their pairwise interactions were analyzed at the next layer. The network is built up layer by layer during training until the stopping criterion based on the “early-stopping” strategy is achieved. Whenever the validation set's performance is reduced at the next layer, the output of the current layer's best neuron was selected as the output of the entire GMDH network. The presented algorithm was used for the binary classification problems. For the extended multi-class problem, the Macro-averaged F_1_-score^82^ was used instead of the MCC as the fitness function.

### Comparison with the state-of-the-art

Other classifiers, namely linear discriminant analysis (LDA), multilayer perceptron (MLP), and supported vector machines (SVM), were used for comparison. LDA is a base classifier used to identify whether the classes could be accurately identified using linear boundaries. SVM, on the other hand, constructs a hyperplane in a high-dimensional space. The nonlinear SVM with the radial basis function (RBF) kernel was used in our study. We tuned the RBF kernel radius and the soft-margin parameter using the method proposed by Wu and Wang^83^. MLP, a feed-forward fully-connected artificial neural network (ANN) model, maps a set of inputs onto an output. In our study, ten neurons with the sigmoid active function and one hidden layer were used. The parameters of the network were tuned on the validation set.

### The validation framework

In our study, threefold cross-validation with stratified sampling^84^ was used. The same test folds were used for different classifiers. In the GMDH and MLP classifiers, 75% of the training set was used for estimation, while 25% was used for validation. The performance indices in Supplementary Table S2 were reported for different classifiers. True Positive, False Positive, True Negative, and False Negative were calculated by comparing the classifiers' results and the gold standard in four classification problems (i.e., depression symptom, mild-to-moderate emotional symptoms, worriedness symptom, and finally psychiatric symptoms). The interpretation of the reference intervals of the indices AUC, K(C), MCC, and DP^85^ was listed in Supplementary Table S3. Moreover, following the STARD guideline^86^, the CI 95% of the performance indices were reported for the cross-validated confusion matrices. The performance of the proposed GMDH algorithm on the three-class extended problem was assessed based on the Macro- and -Micro averaged indices presented by Sokolova and Lapalme^82^.

### Statistical analysis

In this paper, subjects with complete information were used in the analysis. Only for comparison^87^ (Table 5), Multivariate Imputation by Chained Equations (MICE) R-package^88^ was used to impute the ordinal data of the enrolled subjects. All variables were reported, as the frequency and percentage, since they were categorical. The χ^2^ analysis was used to compare the categorical variables in their categories. The Spearman's rho is used as the correlation coefficient between interval-ordinal pairs. Kendall's $\tau_{b}$ was used as the correlation coefficient between ordinal-ordinal pairs. The phi coefficient and rank-biserial correlation coefficient were used for the binary-binary and binary-ordinal association, respectively^40^. The Cochran's Q test with McNemar's post hoc test for pairwise comparison with Bonferroni correction was used to compare different classifiers' performance. A significance level of 0.05 was used in our analysis. MATLAB version 8.6 (The MathWorks Inc., Natick, MA, USA) was used for classification, while R version 4.0.0 (R Core Team (2020), https://www.R-project.org/) was used for data imputation. The statistical analysis was performed using the SPSS statistical package, version 18.0 (SPSS Inc., Chicago, IL, USA).

## Supplementary Information

Supplementary Tables.

## Data Availability

The datasets generated during and/or analyzed during the current study are available from the corresponding author on reasonable request.
