# Supplementary material for: Classification of psychiatric symptoms using deep interaction networks: the CASPIAN-IV study
Source: Sci Rep. 2021 Aug 3;11:15706. doi: 10.1038/s41598-021-95208-y (PMC8333323; doi:10.1038/s41598-021-95208-y)
Supplement: Supplementary file 1 — Supplementary Tables. [file 41598_2021_95208_MOESM1_ESM.docx]

**Classification of psychiatric symptoms using deep interaction networks: the CASPIAN-IV Study**

Hamid Reza Marateb^1,2^, Zahra Tasdighi^3¶^, Mohammad Reza Mohebian^4¶^, Azam Naghavi^5¶^, Moritz Hess^6^, Mohammad Esmaiel Motlagh^7^, Ramin Heshmat^8^, Marjan Mansourian^9,10,*^, Miguel Angel Mananas^11,12^, Harald Binder^13^, and Roya Kelishadi^14,*^

^1^Biomedical Engineering Department, Engineering Faculty, University of Isfahan, 81746-73441 Isfahan, Iran; email: [h.marateb@eng.ui.ac.ir](mailto:h.marateb@eng.ui.ac.ir)

^2^Biomedical Engineering Research Centre (CREB), Automatic Control Department (ESAII), Universitat Politècnica de Catalunya-Barcelona Tech (UPC), Barcelona, 08028 Spain; email: [hamid.reza.marateb@upc.edu](mailto:hamid.reza.marateb@upc.edu)

^3^Epidemiology and Biostatistics Department, Health School, Isfahan University of Medical Sciences, 81746-73461 Isfahan, Iran; email: [zahraa.tasdighi@gmail.com](mailto:zahraa.tasdighi@gmail.com)

^4^Department of Electrical and Computer Engineering, University of Saskatchewan, S7N 5A9 Saskatoon, SK, Canada; email: [mom158@usask.ca](mailto:mom158@usask.ca)

^5^Department of Counseling, Faculty of Education and Psychology, University of Isfahan, Azadi Sq, Isfahan 8174673441, Iran; email: [az.naghavi@edu.ui.ac.ir](mailto:az.naghavi@edu.ui.ac.ir)

^6^Faculty of Medicine and Medical Center – University of Freiburg, 79104 Freiburg, Germany; email: [hess@imbi.uni-freiburg.de](mailto:hess@imbi.uni-freiburg.de)

^7^Department of Pediatrics, Ahvaz Jundishapur University of Medical Sciences, 61357-15794 Ahvaz, Iran; email: [memotlagh@ajums.ac.ir](mailto:memotlagh@ajums.ac.ir) , [dr.motlagh.ms@gmail.com](mailto:dr.motlagh.ms@gmail.com)

^8^Chronic Diseases Research Center, Endocrinology and Metabolism Population Sciences Institute, Tehran University of Medical Sciences, Tehran, Iran; email: [rheshmat@tums.ac.ir](mailto:rheshmat@tums.ac.ir)

^9^Biomedical Engineering Research Centre (CREB), Automatic Control Department (ESAII), Universitat Politècnica de Catalunya-Barcelona Tech (UPC), Barcelona, 08028 Spain; email: [marjan.mansourian@upc.edu](mailto:marjan.mansourian@upc.edu)

^10^Pediatric Cardiovascular Research Center, Isfahan Cardiovascular Research Institute, Isfahan university of medical sciences, 81746-73461 Isfahan, Iran; email: [j_mansourian@hlth.mui.ac.ir](mailto:j_mansourian@hlth.mui.ac.ir)

^11^Biomedical Engineering Research Centre (CREB), Automatic Control Department (ESAII), Universitat Politècnica de Catalunya-Barcelona Tech (UPC), Barcelona, 08028 Spain; email: [miguel.angel.mananas@upc.edu](mailto:miguel.angel.mananas@upc.edu)

^12^Biomedical Research Networking Center in Bioengineering, Biomaterials, and Nanomedicine (CIBER-BBN), 28029 Madrid, Spain.

^13^Faculty of Medicine and Medical Center – University of Freiburg, 79104 Freiburg, Germany; email: [binderh@imbi.uni-freiburg.de](mailto:binderh@imbi.uni-freiburg.de)

^14^Pediatrics Department, Child Growth and Development Research Center, Research Institute for Primordial Prevention of Non-communicable Disease, Isfahan University of Medical Sciences, Isfahan, Iran; email: [kelishadi@med.mui.ac.ir](mailto:kelishadi@med.mui.ac.ir)

^¶^These authors equally contributed to the work.

*: Correspondence

Biomedical Engineering Research Center (CREB), Building H, Floor 4, Av. Diagonal 647, 08028 Barcelona, Spain.

Phone: +34 (658) 085-138

Fax: +34 (93) 401-1676

Pediatrics Department, Child Growth and Development Research Center, Research Institute for Primordial Prevention of Non-communicable Disease, Isfahan University of Medical Sciences, Hezar Jerib street, 8174673461 Isfahan, Iran.

Phone: +98 (31) 3792-3071

**Supplementary Table S1**. List of questions related to the predictors of psychiatric problems based on the Persian version of the Global School-based student Health Survey (GSHS) questionnaire of the CASPIAN IV study.

| Question No. | Psychiatric problems | Responses (scoring) |
| --- | --- | --- |
| 1 | During the past 6 months how often have you felt worthless? | 1. Approximately every day (considered as yes)  2. More than once per week (considered as yes)  3. Approximately every week (considered as yes)  4. Approximately every month (considered as no)  5. Rarely or never (considered as no) |
| 2 | During the past 6 months how often have you got angry too soon? | 1. Approximately every day (considered as yes)  2. More than once per week (considered as yes)  3. Approximately every week (considered as yes)  4. Approximately every month (considered as no)  5. Rarely or never (considered as no) |
| 3 | During the past 6 months how often have you felt anxious? | 1. Approximately every day (considered as yes)  2. More than once per week (considered as yes)  3. Approximately every week (considered as yes)  4. Approximately every month (considered as no)  5. Rarely or never (considered as no) |
| 4 | During the past 6 months how often have you had a bad sleep? | 1. Approximately every day (considered as yes)  2. More than once per week (considered as yes)  3. Approximately every week (considered as yes)  4. Approximately every month (considered as no)  5. Rarely or never (considered as no) |
| 5 | During the past 6 months how often have you felt dizzy or confused? | 1. Approximately every day (considered as yes)  2. More than once per week (considered as yes)  3. Approximately every week (considered as yes)  4. Approximately every month (considered as no)  5. Rarely or never (considered as no) |
| 6 | During the past 12 months, have you had two complete weeks of sadness preventing from your routine activities? | 1.Yes 2. No |
| 7 | During the past 12 months, how often have you been so worried about something that you could not sleep at nights? | 1.Never (considered as no)  2. Rarely (considered as no)  3. Sometimes (considered as no)  4. Mostly (considered as yes)  5. Always (considered as yes) |

**Supplementary Table S2**. The performance measures of the computer-aided diagnosis system

| $Se=Rl=\frac{TP}{TP+FN}$ | $Sp=\frac{TN}{TN+FP}$ | $Acc=\frac{TP+TN}{TP+TN+FP+FN}$ |
| --- | --- | --- |
| $\Pr=\frac{TP}{TP+FP}$ | $FA=\alpha=1-Sp$ | $Power=1-\beta=Se$ |
| $F_{1}S=\frac{TP}{TP+0.5\times\left( FP+FN \right)}$ | $AUC=\frac{Se+Sp}{2}$ | $LR^{+}=\frac{Se}{1-Sp}$ |
| $LR^{-}=\frac{1-Se}{Sp}$ | $DOR=\frac{LR^{+}}{LR^{-}}$ | $DP=\left( \sqrt{\frac{3}{\pi}} \right)\times\log\left( DOR \right)$ |
| $MCC=\frac{TP\times TN-FP\times FN}{\sqrt{\left( TP+FP \right)\times\left( TP+FN \right)\times\left( TN+FP \right)\times\left( TN+FN \right)}}$ | $K\left( C \right)=\frac{2\times\left( TP\times TN-FP\times FN \right)}{\left( TP+FP \right)\times\left( FP+TN \right)+\left( TP+FN \right)\times\left( FN+TN \right)}$ | $NPV=\frac{TN}{TN+FN}$ |

TP: True Positive; FN: False Negative; FP: False Positive; TN: True Negative; Se: sensitivity; Rl: recall; Sp: specificity; FA: false alarm; Acc: accuracy; Pr: precision; F1S: F1-Score; AUC: area under the receiver operating characteristic (ROC) curve; LR: likelihood ratio; DOR: diagnosis odds ratio; MCC: Matthews correlation coefficient; DP: discriminant power; K(C): Cohen's kappa coefficient; NPV: Negative Predictive Value.

**Supplementary Table S3.** The interpretation of the reference intervals of the performance indices used in this study.

| Performance Index | Property | The interpretation of the reference intervals |
| --- | --- | --- |
| AUC ROC | Balanced diagnosis accuracy | [0.7,0.8) good  [0.8,0.9) very good  [0.9,1.0] excellent |
| Kappa | Class labeling agreement rate | <0.40 poor  [0.40,0.75) fair to good  [0.75,1.00] excellent |
| MCC | Correlation between predicted and observed class labels | [0.0, 0.3) negligible  [0.3, 0.5) low  [0.5, 0.7) moderate  [0.7, 0.9) high  [0.9, 1.0] very high  (-1.0,0.0) negative (i.e. disagreement) |
| DP | Discriminant power | <1 poor  [1,2) limited  [2,3) fair $\geq$  3 good |

AUC ROC: area under the receiver operating characteristic curve; Kappa: Cohen's kappa coefficient; MCC: Matthews correlation coefficient; DP: discriminant power.
